# Connexin 43: A Target for the Treatment of Inflammation in Secondary Complications of the Kidney and Eye in Diabetes

**DOI:** 10.3390/ijms23020600

**Published:** 2022-01-06

**Authors:** Chelsy L. Cliff, Bethany M. Williams, Christos E. Chadjichristos, Ulrik Mouritzen, Paul E. Squires, Claire E. Hills

**Affiliations:** 1 Joseph Banks Laboratories, School of Life, Sciences University of Lincoln, Lincoln LN6 7DL, UK; CCliff@lincoln.ac.uk (C.L.C.); Bewilliams@lincoln.ac.uk (B.M.W.); PSquires@lincoln.ac.uk (P.E.S.); 2 National Institutes for Health and Medical Research, UMR-S1155, Batiment Recherche, Tenon Hospital, 4 Rue de la Chine, 75020 Paris, France; christos.chadjichristos@inserm.fr; 3 Ciana Therapeutics, Ole Maaloes Vej 3, 2200 Copenhagen N, Denmark; info@cianatx.com

**Keywords:** diabetes, complications, diabetic nephropathy, diabetic retinopathy, connexin 43, hemichannels, hemichannel blockers, inflammation, purinergic, adenosine triphosphate

## Abstract

Of increasing prevalence, diabetes is characterised by elevated blood glucose and chronic inflammation that precedes the onset of multiple secondary complications, including those of the kidney and the eye. As the leading cause of end stage renal disease and blindness in the working population, more than ever is there a demand to develop clinical interventions which can both delay and prevent disease progression. Connexins are membrane bound proteins that can form pores (hemichannels) in the cell membrane. Gated by cellular stress and injury, they open under pathophysiological conditions and in doing so release ‘danger signals’ including adenosine triphosphate into the extracellular environment. Linked to sterile inflammation via activation of the nod-like receptor protein 3 inflammasome, targeting aberrant hemichannel activity and the release of these danger signals has met with favourable outcomes in multiple models of disease, including secondary complications of diabetes. In this review, we provide a comprehensive update on those studies which document a role for aberrant connexin hemichannel activity in the pathogenesis of both diabetic eye and kidney disease, ahead of evaluating the efficacy of blocking connexin-43 specific hemichannels in these target tissues on tissue health and function.

## 1. Introduction

Impacting almost 10% of adults, diabetes is a global healthcare concern that affects an estimated 463 million people worldwide. With the prevalence of diabetes expected to rise to 700 million people by 2045 [1], it is not the treatment of the disease itself, but the management of associated secondary complications which poses the greatest threat to our healthcare system [2]. Disease complications in diabetes can be categorised as either macrovascular or microvascular, with the former associated with coronary artery disease [3], peripheral arterial disease [4], and stroke [5], whilst microvascular complications include nephropathy [6,7,8], retinopathy [9,10] and impaired wound healing [11,12]. In the early stages of disease progression, management focuses on regulation of blood pressure and maintenance of good glycaemic control [13]. However, for many, deterioration of good health is inevitable, with kidney failure, loss of vision or circulatory problems, contributed to by comorbidities (e.g., hypertension, obesity, cardiovascular disease) and health inequalities [14]. In the absence of a definitive treatment for these conditions, new therapeutic approaches are urgently required.

In diabetes, complications develop in response to sustained hyperglycaemia and low-grade systemic inflammation, the latter of which is heightened in type 2 diabetes mellitus (T2DM), where coupled with obesity, increased adipose tissue secretes inflammatory mediators that exacerbate a state of pre-existing inflammation [15,16,17,18]. There is a strong association between microvascular complications in patients with T2DM, and individuals who present with diabetic nephropathy often experience higher incidence of retinopathy compared to patients without any diabetes-related kidney issues [19,20,21,22]. Similarly, individuals exhibiting diabetic retinopathy appear more susceptible to the onset of kidney problems [23]. These findings suggest a ‘common pathway’ representative of systemic microvascular damage and chronic inflammation that, secondary to diabetes, leads to a progressive loss of tissue function. Recent retinopathy and nephropathy studies strongly suggest that blocking expression and/or function of small transmembrane proteins called connexins under pathophysiological conditions, may significantly dampen the inflammatory response that drives disease progression across these and other age associated pathologies, e.g., obesity [24,25], Alzheimer’s disease [26,27] and osteoarthritis [28].

**Figure 1:**
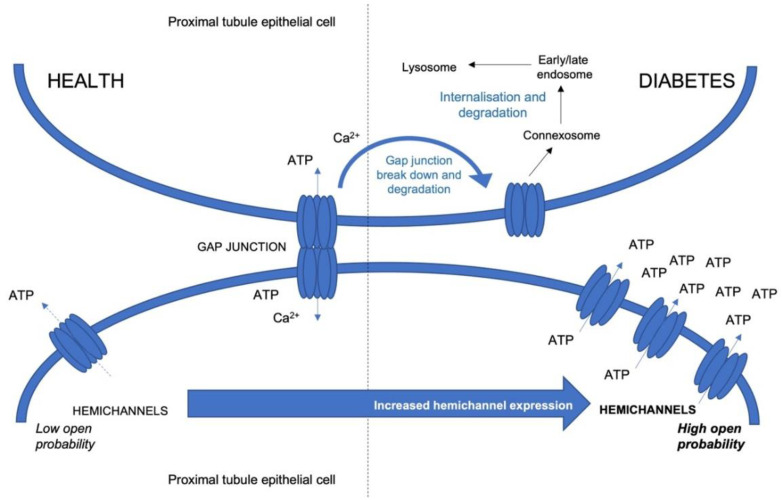
A schematic highlighting changes in hemichannel activity in health and diabetes. During injury, gap junctions break apart. These broken channels undergo endocytosis, assemble into a double membrane structure termed a connexosome and then experience endosome sorting prior to transportation to lysosomes for degradation [53]. These events are paralleled by an upregulation of hemichannel activity and number, leading to an increase in release of molecules, including ATP, causing downstream inflammation and fibrosis via purinergic signalling.

Connexins are a family of membrane bound proteins involved in the transfer of small molecules and ions between two cells (gap junctions) and between cells and their immediate environment (hemichannels), highlighted in Figure 1. Nomenclature is dictated by molecular weight [29], with connexin 43 (Cx43) the most abundant in humans [30]. Composed of one intracellular and two extracellular loops, and an N- and C-terminus [31], they oligomerise into hexameric structures called connexons and are delivered to the plasma membrane in vesicles that transit along a secretory pathway [32,33]. When neighbouring cells align, connexons dock to form a continuous gap junction, establishing a direct route for cell-cell communication that allows cells to synchronise their activity [34,35,36]. Whilst gap junction activity maintains cellular function under physiological conditions undocked connexons, referred to as hemichannels, are typically linked with pathophysiological stimuli, such as oxidative stress [37] and inflammation [35,38]. Dysregulation of hemichannel function is associated with chronic diseases, including deafness [39], brain ischaemia [40] and chronic pain [41,42]. The role of hyperglycaemia in regulating connexin expression [43,44], gap junction communication [45] and hemichannel activity [46,47,48] is well documented [49], and of the 21 isoforms known to be expressed within the human body, Cx43 has been strongly linked to the pathogenesis of multiple secondary complications of diabetes [43,50,51,52]. In this article, we review a role for Cx43 hemichannels in chronic inflammation and microvascular complications of diabetic nephropathy and retinopathy, ahead of exploring the therapeutic potential of hemichannel blockers in preventing disease progression.

## 2. Targeting Inflammation in Microvascular Complications of Diabetes

Instrumental to the pathogenesis of diabetes and its complications, targeted anti-inflammatory therapy has been suggested for both prevention and treatment of diabetes and has been extensively reviewed [54,55,56]. Known to underpin disease progression across multiple age associated conditions e.g., diabetes [55], obesity [25] and age-related macular degeneration [57], recent attention has focussed on the design of pharmacological compounds that block key inflammatory candidates, such as the nod-like receptor protein 3 (NLRP3) inflammasome (e.g., MCC950) [58], changes in cell phenotype (e.g., senolytics) [59] or cell function (e.g., sodium-glucose co-transporter-2 inhibitors: SGLT2i) [60,61,62]. 

The NLRP3 inflammasome has been referred to as the ‘grumpy old man of inflammation’ [63] and is linked to a variety of inflammatory conditions including atherosclerosis [64], Alzheimer’s disease [27], inflammatory bowel disease [65], and non-alcoholic steatohepatitis [66]. It is upregulated in immune and epithelial cells across different tissue types, where activation culminates in secretion of pro-inflammatory mediators, interleukin-1β (IL1β) and interleukin-18 (IL18). In turn, these activate tumour necrosis factor-alpha (TNFα) and interleukin-6 (IL6), both of which exhibit increased serum levels with age and disease and mediate inflammation/fibrosis in multiple secondary complications of diabetes [67,68,69,70,71]. Since chronic inflammatory conditions are amplified and perpetuated by the inflammasome pathway, it is not surprising that blocking the NLRP3 inflammasome directly (e.g., MCC950) alleviates inflammation across multiple age-associated morbidities [60,68,72].

Despite these encouraging observations, blanket blockade of a complex integral part of the innate immune response has been met with concern. Activated by both damage-associated molecular patterns (DAMPs) and pathogen associated molecular patterns (PAMPs), the NLRP3 inflammasome mediates both sterile and non-sterile inflammation [73]. Consequently, whilst inhibition of NLRP3 inflammasome may protect against sterile inflammation induced by endogenous noxious stimuli, this could render individuals susceptible to injury where PAMP-associated microbial infection fails to elicit a response [74,75]. Nevertheless, with the NLRP3 having been identified as a key mediator of inflammation in over 80 different models of injury [76,77,78,79,80], it is not surprising that various compounds have entered clinical trials, e.g., Inzomelid (NCT04015076), IFM-2427 (DFV890) (NCT04382053) and Dapansutrile (OLT1177) (NCT04540120) [81]. Despite this, a drug which successfully targets NLRP3 is yet to reach its primary endpoint, an observation compounded by our lack of knowledge of its structure and potential binding sites [82]. Consequently, interventions to target downstream mediators e.g., IL1β and TNFα, have received considerable attention. Canakinumab (ACZ885, Ilaris) is a recombinant human monoclonal antibody that selectively inhibits IL1β receptor binding and demonstrated positive primary outcomes in the Canakinumab Anti-inflammatory Thrombosis Outcome Study (CANTOS) [83]. It subsequently became licensed for the treatment of rare inflammatory conditions, including juvenile arthritis [84]. However, its efficacy proved disappointing in the treatment of inflammation in diabetic retinopathy (NCT01589029), Type I Diabetes Mellitus (T1DM) (NCT00947427, [85]) and atherosclerosis (NCT00900146, [86]), an effect potentially linked to increased infection rates and sepsis [87]. Similar efforts to target TNFα include compounds that contain either receptor fusion proteins (etanercept) [88], which suppress the physiological response to TNFα, or monoclonal antibodies (golimumab, infliximab, adalimumab and certolizumab pegol), all of which have met mixed success [89].

Whilst evident that there is much to learn in our quest to develop new interventions that successfully (a) target sterile inflammation and (b) do so in the absence of serious side effects, recent FDA approval of SGLT2 inhibitors has perhaps been the most significant step forward in managing and improving outcomes in patients with nephropathy [90] and cardiovascular disease [91]. By blocking sodium glucose co-transport and reducing blood glucose levels, SGLT2i demonstrate improved renal and cardiovascular outcomes in patients with T2DM and diabetic nephropathy [92,93,94]. Whilst initial protection is thought to stem from a decrease in glomerular hyperfiltration, several studies demonstrate that SGLT2i confer protection via suppression of inflammation and fibrosis, albeit the widespread mechanisms remain to be fully elucidated [95,96,97]. However, with prescription targeted to individuals with T2DM as opposed to T1DM and potential side effects that include ketoacidosis [98], increased risk of amputation [99], and increased genitourinary tract infection [100], SGLT2i are not a one size fits all. As an alternative approach, Cx43 hemichannel blockers are a class of drugs which include Gap19 [101] and Tonabersat [102,103]. They bind to, and close hemichannels to prevent the release of numerous DAMPs, including ATP (for a more detailed review of how these peptides work we refer the reader to King et al. as published in this special issue) [104]. In the presence of DAMPs, the NLRP3 complex is activated and elicits an inappropriate inflammatory response, stimulating and activating via local paracrine mediated signalling, both infiltrating immune cells and resident fibroblasts [50,78,105,106]. On this basis alone, it is not hard to understand why connexin hemichannel blockers are increasingly championed as an effective therapeutic strategy in inhibition of sterile inflammation in disease.

## 3. Cx43 Hemichannel Blockers and Treatment of Inflammation in Diabetes and Its Secondary Complications

With evidence that Cx43 hemichannel mediated communication contributes to the pathogenesis and progression of tissue damage in secondary complications of diabetes [49,107,108], drugs that target Cx43 hemichannels have been identified as potential anti-inflammatory therapies [109,110]. Compounds of interest include Peptide 5, known to bind to the second extracellular loop of Cx43 [111]; Gap26, which mimics the first extracellular loop of Cx43 [112]; and αCT1, a Cx43-based peptide [113]. Their mechanism of action, along with models in which they have been trialled are summarised in Table 1. These compounds specifically inhibit Cx43 hemichannel opening [114] and have demonstrated efficacy in preventing the release of tissue damage inducing signals and thus alleviating downstream inflammation and fibrosis in secondary complications of diabetes [105], including diabetic nephropathy [108]. 

## 4. Cx43 Hemichannels and Treatment of Inflammation in Diabetic Kidney Disease

Diabetic nephropathy is widely regarded as a glomerular disease, where proteinuria is the predominant early clinical marker [140]. Signs of glomerular injury include podocyte damage and effacement [141,142], crescent formation [143], basement membrane thickening [144], macrophage infiltration [145] and inflammation [146]. Early studies evaluating a link between connexins and glomerular damage observed increased Cx43 expression in both biopsies from injured human glomeruli and in the nephrotoxic glomerulonephritis (NTS-GN) murine model of chronic kidney disease [147]. The NTS-GN models exhibits a similar presentation to the streptozotocin (STZ) mouse model of T1DM, with histological and functional studies reporting that these mice develop glomerulosclerosis, inflammation, fibrosis, and albuminuria [148]. In NTS-GN mice, up-regulation of Cx43 occurs via increased binding of activated protein-1 (AP-1) transcription factors, phosphorylated (p)-cellular (c) JUN, p-signal transducer, and activator of transcription-1 (STAT1) to the Cx43 promoter [147]. Furthermore, in mice treated with a Cx43 specific antisense oligodeoxynucleotide or in the heterogenous Cx43^+/−^ mouse induced with NTS-GN, proteinuria, blood urea nitrogen (BUN) and serum creatinine levels are reduced [148]. Similar observations were also reported in the STZ-induced rat when treated with Cx43 small interfering ribonucleic acid (siRNA) [149]. This protection may stem from impaired autophagy, an intracellular degradation mechanism which removes/recycles dysfunctional or unnecessary cellular components to ensure efficient health and function of the cell [149]. Mediated through activation of mammalian target of rapamycin (mTOR) signalling, high glucose treated mouse podocytes (MPC5) in which Cx43 expression was reduced via transient transfection with siRNA, exhibit reduced mTOR activation, impaired autophagic flux and decreased podocyte injury [149]. Paracrine mediated purinergic signalling has also been linked to Cx43 induced podocyte injury, with transforming growth factor beta 1 (TGFβ1) treated mouse E.11 podocytes, co-incubated with Cx43 specific blocking peptide Gap26 and purinergic receptor blocker suramin, exhibiting attenuated cytoskeletal reorganisation, improved morphology and a decrease in apoptosis compared to TGFβ1 alone [147].

Although a glomerular disease in origin, advanced stages of nephropathy are characterised by severe tubule interstitial inflammation and fibrosis [150]. Work within our laboratories links altered Cx43 expression to tubule injury in both in vitro [108,135,151,152] and in vivo [108] models of disease [153]. Initial observations identified an approximate 5-fold increase in Cx43 expression in biopsy material from individuals with diabetic nephropathy compared to healthy control [151], whilst paired-patch electrophysiology and ATP biosensing suggested that this increased expression was paralleled by diminished gap-junction intercellular coupling (GJIC) and increased hemichannel mediated ATP release [151]. With intercellular adhesion a pre-requisite for gap junction formation, AFM-single cell force spectroscopy [154] determined that this loss of direct cell coupling paralleled the reduction of E-Cadherin mediated cell adhesion [155], an effect significantly blunted by co-incubation with the P2X7 receptor (P2X7R) antagonists Suramin, A438079 or A804598 [108,156]. Previous studies link P2X7R activation to macrophage and extracellular matrix deposition in both in vitro models of diabetic kidney disease [108,157] and in STZ-induced diabetic mice [157], whilst we recently observed increased P2X7R expression in renal biopsy from people with diabetic nephropathy and in the unilateral ureteral obstruction (UUO) mouse model [108]. The UUO is a model of advanced interstitial inflammation and fibrosis which recapitulates late-stage damage observed in the diabetic kidney, irrespective of the initiating stimuli [158]. It is widely used for mechanistic studies in all forms of advanced CKD [158]. Despite our knowledge of a role for P2X7R activation in disease pathogenesis, attempts to target P2X7 have been relatively unsuccessful, potentially due to the genetic variability of the human P2X7 receptor which can lead to altered pharmacodynamic responses [159,160]. Consequently, having identified that impaired gap junction coupling is paralleled by increased hemichannel mediated ATP release [151], combined with evidence that elevated ATP and sustained P2X7R are linked to onset and progression of inflammation and fibrosis in multiple tissue types, we assessed a role for both Cx43 and P2X7R activation in disassembly of the adherens and tight junction complex in both TGFβ1 treated human primary tubule cells co-incubated with P2X7R inhibitors A438079 and A804598 and in the Cx43^+/−^ UUO mouse model [108]. Blocking the P2X7R significantly blunted the TGFβ1 evoked change in adherens (E-Cadherin, N-Cadherin) and tight junction proteins (Claudin-2 and Zona Occludins (ZO-1)), whilst restoring both cell adhesion and paracellular permeability [107]. Not surprisingly, these TGFβ1-induced effects were significantly diminished when cells were co-incubated with Apyrase, an ATP-diphosphohydrolase that catalyses the sequential hydrolysis of ATP to ADP, then AMP and adenosine, suggesting a downstream role for ATP in mediating the actions of TGFβ1. The origin of this signal is further supported by our recent studies in the Cx43^+/−^ UUO mouse where disassembly of the adherens (e.g., E-cadherin) and tight (e.g., ZO-1) junction complexes were significantly blunted as compared to wild-type control [108]. Whilst collectively these studies support a role for Cx43 and downstream purinergic signaling in tubular injury, understanding how this protection is conferred is instrumental if wanting to target this communication through pharmacological intervention.

In contrast to the observations above, Sun et al. recently suggested that Cx43 expression is downregulated in diabetic kidney disease and that overexpression of Cx43 using short hairpin RNAs attenuates renal fibrosis and reduces epithelial-to-mesenchymal transition (EMT) in a carboxyl-terminal signal transduction-dependent manner in leptin receptor-deficient type 2 diabetic (db/db) mice and in rat kidney NRK-52E cells treated with high (30 mM) glucose [161]. They attributed this non-channel dependent effect to regulation of the sirtuin-1 hypoxia inducible factor-1alpha (SIRT1-HIF-1ɑ) signalling pathway and have more recently suggested that the protective effects of Cx43 are associated with ubiquitin-specific protease 9X (USP9X/FAM) mediated de-ubiquitination [161]. Whilst the implications of this altered Cx43 expression for cell communication remains to be reported in these models, combined evidence from other studies suggest that blocking Cx43 hemichannels through mimetic peptides may represent a novel approach in targeting inflammation and fibrosis in multiple tissue types [108,113,162,163].

Peptide 5 is a connexin peptidomimetic that mimics a portion of the 2nd extracellular loop of Cx43 [111] and has proven effective in blocking Cx43 hemichannels and preventing ATP release in multiple models of injury when delivered intraocularly [126], into cerebrospinal fluid [164] and systemically [165]. Studies confirm target applicability and specificity and yield similar and significant benefits across different injury models [108,126,164,165,166]. Our recent findings determined that elevated levels of TGFβ1 increase Cx43 hemichannel mediated ATP release [151], an effect which drives P2X7R mediated phenotypic changes linked to initiation of partial EMT in the proximal region of the kidney [108,164]. Co-incubation of TGFβ1 treated human proximal tubule epithelial cells (hPTECs) with Peptide 5, successfully blocked hemichannel mediated carboxyfluorescein dye uptake and real time ATP release, the impact of which was evidenced by restoration of expression of adherens and tight junction proteins in injured cells [108].

Instrumental to cell adhesion and maintenance of polarity, disassembly of cell junction complexes is linked to partial EMT, events which predispose inflammation and fibrosis [167], the latter of which is contributed to by extracellular matrix (ECM) deposition [152]. With collagen I increased in the interstitium of UUO mice, an effect lessened in the Cx43^+/−^ model [168], we hypothesized that a modified microenvironment may elicit phenotypic changes via increased Cx43 mediated hemichannel ATP release. Consequently, we observed that TGFβ1 treated human kidney cells bond with increased affinity to collagen I via integrin isoform α2β1, an interaction which shifted the cell phenotype to one of increased expression of integrin linked kinase, *N*-cadherin, fibronectin and collagen IV as compared to cells uncoated control. Interestingly, co-incubation of TGFβ1 treated cells with Peptide 5 significantly blocked the increase in hemichannel mediated dye uptake and ultimately restored expression of markers of tubular injury to levels representative of control cells cultured on plastic. Moreover, Peptide 5 blocked TGFβ1 induced secretion of collagen I [168], corroborating in vivo data in the Cx43^+/−^ mouse [168] and highlighting the existence of a potential feedback loop in which aberrant Cx43 hemichannel mediated ATP release increases collagen I secretion and deposition, the latter of which perpetuates tubular injury via a Cx43 hemichannel mediated mechanism.

Building on our published observations with Peptide 5, we recently assessed the efficacy of Danegaptide in conferring protection in an in vitro model of tubular injury. A Cx43 gap junction modifier [133,134], we reported that Danegaptide was also able to block hemichannel mediated dye uptake, ATP release and consequently TGFβ1 induced changes in markers of tubular injury e.g., E-Cadherin and N-Cadherin and fibrosis e.g., collagen-I, collagen-IV and fibronectin in human primary tubule epithelial cells (hPTECs) [135]. Furthermore, based on evidence that the Cx43^+/−^ UUO mouse presents with decreased fibroblast activation and diminished macrophage infiltration as compared to wild type UUO control [168], we employed proteome profiler arrays to screen for the expression profile of 125 inflammatory cytokines in TGFβ1 treated human primary proximal tubule cells in the presence/absence of Danegaptide [135]. Soluble chemokines, adhesion molecules and growth factors recruit and activate infiltrating immune cells and resident fibroblasts to mediate inflammation and fibrosis in the diabetic kidney [135]. However, little is known about the switch that triggers release of these chemotactic signals or whether blocking this switch has implications for heterotypic cell communication. Whilst we are yet to fully understand the role of Cx43 hemichannel activity in these paracrine mediated events, we observed that Danegaptide significantly blocked Cx43-mediated ATP release in tubular epithelial cells to negate secretion of many inflammatory mediators, including chemokines, monocyte chemoattractant protein (MCP1), Regulated upon Activation, Normal T Cells Expressed and presumably Secreted (RANTES; involved in macrophage infiltration [169,170]), inflammatory interleukins (IL6 and IL1β) and adipokine adiponectin (associated with macrophage-to-myofibroblast differentiation [171]). In support of our in vitro data and Cx43^+/−^ UUO mouse [108], work by Abed et al. demonstrated that the number of primary monocytes which adhere to an activated mouse endothelial cell monolayer is reduced in endothelial cells co-incubated with Gap26 [168]. The findings highlight the tantalising therapeutic potential of targeting Cx43 hemichannel activity in diabetic nephropathy and other forms of CKD. It remains to be resolved how blocking Cx43 confers protection in vivo, whilst further research is required to assess the efficacy of Cx43 mimetic peptides in a clinical setting.

## 5. The Therapeutic Potential of Blocking Cx43 in Diabetic Retinopathy

Diabetic retinopathy affects around one third of people with diabetes and is the primary contributor to blindness in the working age population [172], often resulting in sight loss as a consequence of diabetic macular oedema, haemorrhage or retinal detachment [173]. This is due to increased cell apoptosis, vascular permeability and disruption of retinal homeostasis [173]. Categorised into two clinical stages of disease, diabetic retinopathy initially presents as a non-proliferative form, characterised by inflammation, hypertrophy, oedema, capillary breakdown, ischemia, and loss of microvascular endothelium integrity leading to abnormal blood-retinal barrier (BRB) permeability [9]. The subsequent decrease in blood flow and nutrient supply drives progression to the proliferative stage where increased blood vessel formation leads to haemorrhage and scar tissue formation [9]. These pathologies can cause detachment of the retina resulting in severe or complete blindness [9].

Loss of vision in retinopathy is associated with breakdown of the retinal pigment epithelium (RPE), where periods of sustained hyperglycemia drive inflammation and apoptosis through increased secretion of key inflammatory mediators, growth factors and hypoxia-inducible factors [174]. This inflammation is believed to actively contribute to associated damage of the retinal vasculature through its ability to trigger apoptosis of RPE cells and promotion of retinal neovascularization. Of the main damage inducing molecules whose activity and expression is known to be upregulated in the diabetic eye, it is the increased secretion of vascular endothelial growth factor (VEGF) which triggers neovascularisation and onset of the proliferative stage of diabetic retinopathy [175]. Coupled with the breakdown of tight junctions between cells of the RPE, disruption to the retinal pigment epithelium allows for these newly developed and fragile blood vessels to push through and leak into the macula. The resulting macular oedema is one of the greatest contributors to sight loss in diabetic retinopathy [176]. Whilst laser treatments [177] and anti-VEGF injections [178] stabilise blood vessels and prevent further neovascularisation respectively, targeting upstream of this RPE breakdown and inflammation is a major focus in the field. Tackling the condition in its early stages, and thus preventing transition of the non-proliferative to proliferative stage, will not only improve patient outcomes but reduce the socioeconomic burden of this disease.

In targeting this damage, it is important to understand how it manifests itself. Interestingly, the series of events which drive proliferative diabetic retinopathy are of a similar aetiology to those which we see in late-stage diabetic kidney disease [23]. As with onset and progression of tubulointerstitial fibrosis [167], the breakdown of the retinal pigment epithelium is associated with disassembly of junction proteins, namely ZO-1, E-cadherin, β-catenin and occludin and ultimately induction of EMT [179]. In fact, EMT of RPE cells is considered an initiating trigger in the loss of epithelial integrity and is driven by glucose-evoked changes in TGFβ [180]. Moreover, a recent study by Lyon et al. identified that inflammation coupled with glycaemic damage mediates EMT of the RPE via aberrant Cx43 mediated hemichannel activity [50], whilst Peptide 5 blocked loss of ZO-1 expression and restores RPE permeability as measured by transepithelial resistance [125]. These studies further support the extensive work in the field of connexin biology and ophthalmology, which in recent years has identified a key pathological role for connexin hemichannels in ophthalmological disease [47].

Both in vitro [105] and in vivo [181] models of diabetic retinopathy have been used to evaluate a role for Cx43 mediated communication when treated with IL1ß and TNFα in the presence of high glucose. Using clonal human retinal pigment epithelial cells (ARPE-19) and NOD mice, Mugisho et al. demonstrated that these cells exhibit increased expression of Cx43, an effect exacerbated in the presence of both glucose and inflammation. This increased Cx43 expression was paralleled by enhanced secretion of pro-inflammatory cytokines (interleukin-6, interleukin-18, monocyte chemoattractant protein-1, and intercellular adhesion molecule 1), angiogenic promoting VEGF [181] and downstream extracellular matrix protein collagen-IV [125]. Furthermore, with evidence that these cells release elevated levels of ATP, the authors subsequently determined that these effects were blunted in the presence of Cx43 hemichannel blocker Peptide 5, whilst exogenous application of ATP and restoration of the response further corroborated a role for Cx43 in driving these in vitro observations [105,125]. In vivo Cx43 expression increased in both the Akimba (albeit not the Akita) mouse, whilst increased expression was also observed in donor retinas with confirmed diabetic retinopathy compared to age-matched controls [182]. In addition, and building on their in vitro observations, the team developed an in vivo model of diabetic retinopathy in which pro-inflammatory cytokines, IL1β and TNFα, were injected into the vitreous of NOD mice. Results showed that injecting intravitreal cytokines into these mice induced a host of parameters detrimental to tissue function, including severe vitreous hyper-reflective foci, vessel dilation, oedema microglia upregulation [183]. With previous evidence that Peptide 5 was able to prevent Cx43 mediated vascular leakage and retinal ganglion cell death after retinal ischaemic injury in rats [159], Peptide 5 was administered to these NOD mice and structural and functional parameters recorded. Injection of Peptide 5 significantly improved vessel dilation and beading, reduced sub-retinal fluid accumulation, decreased microglial infiltration into the outer nuclear layer, and decreased expression of both NLRP3 and the adaptor protein ASC, the latter of which suggests a link between Cx43 hemichannels and activation of the inflammasome pathway [182].

Since the NLRP3 inflammasome is an integral mediator of our innate immune response, the link between aberrant Cx43 hemichannel mediated ATP release and activation of the NLRP3 inflammasome was further explored in vitro, where NLRP3 complex assembly, caspase 1 activation and IL1β secretion were blocked in treated ARPE-19 cells co-incubated with Peptide 5 [105]. Similarly, Cx43 hemichannel blocker, Tonabersat (Xiflam) also protected against retinal injury by blocking Cx43 mediated ATP release, NLRP3 inflammasome activation and the release of pro-inflammatory mediators e.g., IL1β, IL6 and VEGF, in both high treated ARPE-19 cells [50] and in organotypic human retinal explants [184]. Blockade of the NLRP3 inflammasome in addition to those events described above dampened the immune response, reduced aneurysm development and restored cell function. In the light damaged albino rat model of diabetic retinopathy when treated with either Peptide 5 [126] or Tonabersat [185] the resultant changes in photoreceptor function and vascular permeability observed were accompanied by a reduced rate of disease progression [185].

Whilst the evidence of a role for Cx43 hemichannels in driving the pathology of diabetic retinopathy is undeniable, loss of tissue function in response to altered GJIC has also been reported. Under conditions of glycaemic injury, Cx43 gap junction coupling is reduced in retinal capillaries from people with diabetes [185,186], rat microvascular endothelial cells [187] and in pericytes [188]. Implications for these changes were associated with endothelial cell apoptosis [187], pericyte death [186,188] and acellular capillary formation [187]. With evidence that Danegaptide confers protection in in vitro models of diabetic nephropathy and diabetic retinopathy [135,188], the dipeptide has specifically been shown to maintain gap junction coupling between endothelial cells despite high glucose stress, as assessed using scrape load dye transfer. In primary rat retinal endothelial cells, Danegaptide modulated a high glucose induced increase in apoptosis and cell permeability [188], thus further highlighting the promising effects in regulating Cx43 mediated communication via modulation of their activity with mimetic peptides.

## 6. Conclusions

Recent statistics from Eurostat suggest that 28% of Europeans will be aged 65yr and older by 2060, with estimates predicting the average UK life expectancy of women to be 91yr and men 88yr by 2030 [134]. With the prevalence of T2DM linked exponentially to the aging process the incidence of diabetes and its secondary complications is expected to rise. Chronic inflammation is a hallmark of retinopathy [189] and nephropathy [190,191], with induction of morphological and phenotypic cell changes linked to upstream activation of the NLRP3 inflammasome [192,193,194,195,196,197,198,199]. In a bid to target chronic inflammation, the recent field of senolytics and NLRP3 inhibitors have taken centre stage [59,67]. However, although promising (e.g., Dasatanib & Quercertin [200]), more information about safety, tolerability and off-target effects of these drugs is required. In addition, targeting the NLRP3 inflammasome (e.g., MCC950 [200]) or downstream IL1β (e.g., Canakinumab [87,200]) has raised concerns over increased susceptibility to pathogenic infection and long-term side effects. Consequently, treatment of inflammation in progressive nephropathy and retinopathy remains an unmet need. Connexin hemichannel blockers represent a promising future therapeutic option in the treatment of nephropathy and retinopathy. Research to date is persuasive and highlights promising beneficial effects of Cx43 inhibition on inflammation, tissue integrity and fibrosis [180,201,202]. However, the field requires further research to determine the effectiveness and efficacy of drugs and the long-term benefits.

## Figures and Tables

**Table 1:** Cx43 hemichannel blockers, mechanism of action and models in which they have been trialled to date.

Hemichannel Blocker/ Therapeutic Agent	Sequence/ Formula	Mechanism of Action	Examples of Models Trialled in	Clinical Trials?
Gap19	KQIEIKKFK Also: Transactivator of transcription (TAT)-Gap19 -YGRKKRRQRRR-KQIEIKKFK Xentry (XG19) -lclrpvGG-KQIEIKKFK	Binds to the intracellular loop of Cx43, whilst not affecting gap junction communication [115]. Exhibits low cell permeability, so is often coupled with TAT which aids transcription or Xentry which is a cell penetrating peptide [116].	Primary mouse cardiomyocytes [117]; Cerebral ischaemia/injury in mice [118]; Primary mouse astrocytes/hippocampal slices (TAT-Gap19) [115]; Immortalised human retinal pigment epithelium cells (ARPE-19)/primary human retinal microvascular endothelial cells (hREMC) (XG19) [116]; Isolated rat hepatocytes [119]; Human gingival fibroblasts [120].	None found.
Gap26	VCYDKSFPISHVR	Originally developed to block gap junction communication [121]. Now shown to also block hemichannels, Gap26 binds to the first extracellular loop of Cx43 [112].	Isolated pig ventricular cardiomyocytes [117]; Cultured microglia, astrocytes and neurons [122].	None found.
Gap27	SRPTEKTIFII	Originally designed for gap junction blockade [121], Gap27 can also block hemichannels by binding to the second extracellular loop of Cx43 [112].	Isolated pig ventricular cardiomyocytes [117]; Primary human corneal epithelial cells in vitro, human corneas ex vivo rat wound healing model in vivo [123]; Adult keratinocytes, juvenile foreskin, human neonatal fibroblasts and adult dermal tissue as models of wound healing [124].	None found.
Peptide 5	VDCFLSRPTEKT	Binds to the second extracellular loop of Cx43, preventing hemichannel opening [111].	Human primary proximal tubule epithelial cells and clonal tubular kidney epithelial cells [108]; Retinal pigment epithelial cells [105,125]; Patch-clamp inflammatory model in mice [102]; Light-damaged albino rat model [126].	None found
Tonabersat (Xiflam)	C₂₀H₁₉ClFNO₄	Able to block gap junctions (at high concentration), this small molecule, a benzopyran derivative can block Cx43 hemichannels at lower doses [50].	Human retinal pigment epithelial cells (ARPE-19) [50]; Rat model of diabetic retinopathy [127].	Phase II clinical trials in migraines-NCT00311662 NCT00534560 NCT00332007
alpha connexin carboxyl terminus 1 (αCT1)	Ant-RPRPDDLEI	Binds to the COOH tail (cytoplasmic terminus) of Cx43 [113], mediating phosphorylation of Cx43 at serine 368 [128]. Has also been shown to affect gap junction remodelling [129].	Rat model corneal wound [130]; Beneficial in a randomised control trial assessing cutaneous scarring [131]; Human biopsy tissue/rat and guinea pig scars [132].	Clinical trials for diabetic foot ulcers as ‘Grannexin gel’ Phase I-NCT02652754 Phase II-NCT02652572 Terminated at phase III May 2020 (NCT02667327)–no safety concerns
Danegaptide (GAP-134)	C_14_H_17_N_3_O_4_	Not fully elucidated. As a gap-junction modifier, it maintains gap junction coupling during cellular stress [133,134], and has been shown to block Cx43 hemichannels in human proximal tubule epithelial cells [135].	Primary human proximal tubule epithelial cells [135]; Rat Retinal Endothelial cells during high glucose stress [134]; Myocardial infarct in pigs [136] and dogs [137]; Atrial fibrillation models in dogs [138,139].	Phase II for myocardial infarction-NCT01977755
